# Mental Health during the Early Stage of the COVID-19 Pandemic: A Hong Kong Study

**DOI:** 10.3390/ijerph19158957

**Published:** 2022-07-23

**Authors:** Pik-Kwan Cheung, Joseph Wu, Wing-Hong Chui

**Affiliations:** 1 Department of Social and Behavioural Sciences, City University of Hong Kong, Kowloon Tong, Kowloon, Hong Kong, China; pkcheung36-c@my.cityu.edu.hk; 2 Department of Applied Social Sciences, The Hong Kong Polytechnic University, Hung Hom, Kowloon, Hong Kong, China; wing-hong.chui@polyu.edu.hk

**Keywords:** mental health, COVID-19, Hong Kong, self-compassion, valence of event, hope

## Abstract

This study addressed the impact on mental health and wellbeing in relation to views of the self, the world, and the future during the early stage of the global novel coronavirus (COVID-19) pandemic outbreak. An online survey battery included the 21-item Depression Anxiety and Stress Scale (DASS-21), Subjective Happiness Scale, Self-Compassion Scale, Adult Hope Scale, and two specifically-written items measuring the valence quality and quantity of the impact of the pandemic. A total of 345 Hong Kong residents (54% females) responded; 17.1%, 24.7%, and 19.7% reported elevated levels of depression, anxiety, and stress, respectively. The prevalence of these issues was lower in this Hong Kong sample than reported in other COVID-19 studies, possibly due to the past experience of the severe acute respiratory syndrome (SARS) outbreak in Hong Kong in promoting the voluntary wearing of masks in public places and introducing social distancing measures during the early first wave of the pandemic. Correlational analyses showed associations between positive views about the self (higher self-compassion), the world (higher positive valence), and the future (more hope) and better mental health and psychological wellbeing (fewer depression, anxiety, and stress symptoms; more happiness). Regression analyses indicated a differential predicting power of the three views on the four selected mental health and psychological wellbeing indicators. Intervention programs to alleviate distress and/or promote better wellbeing should be matched to the specific problems encountered by the sufferers.

## 1. Introduction

On 11 March 2020, the World Health Organization (WHO) officially declared the novel coronavirus to be a global pandemic outbreak and named it COVID-19 [1]. There were 118,000 cases reported in 114 countries and more than 90 percent of these cases were in China, South Korea, Iran, and Italy. Hong Kong is one of the major cities in China, connecting the east and west. The first confirmed COVID-19 case in Hong Kong was on 23 January 2020 and the first wave of the pandemic (from January to May 2020) peaked in late March, with around 1000 cases, according to information from the Centre of Health and Protection [2]. The incidence and mortality (135.5 and 0.5 per 1,000,000) in Hong Kong was the lowest in the world in the first wave of the pandemic (from January to April 2022) [3].

Hong Kong experienced the severe acute respiratory syndrome (SARS) outbreak in 2003, which lasted from March to June, with 1750 reported cases and 286 deaths. The first outbreak was found in Guangdong, China, in November 2002 and was similar to a typical pneumonia. It spread later to other provinces in China, with a total of 5329 confirmed cases and 336 deaths in June 2003. There was some limited outspread to other countries and cities in Asia and the Pacific, such as Singapore, Vietnam, and Taiwan [4]. With the SARS experience, a close eye was kept on the COVID-19 development in Wuhan, China, from the first reported case on 31 December 2019. In the 2003 experience, the medical organizations and healthcare workers in Hong Kong overcame the SARS epidemic within a few months. This meant that the majority of Hong Kong people thought that COVID-19 would be another kind of SARS. Most people living in this city expected that this pandemic would go away sooner or later. It is likely that this collective optimistic frame of reference may have caused Hong Kong people to view the outbreak as less threatening, and they were hopeful for a quick outcome. In addition, during the SARS outbreak, the people learned to recognize that voluntarily wearing face masks and complying with various hygiene regulations enforced by the government showed their willingness to sacrifice individual living quality for the collective common interest of preventing the spread of the virus in the community [2].

The pandemic affected not just the physical health of those who were infected, but also the mental health and wellbeing of the general public. In a meta-analysis examining the prevalence of depression, anxiety, and stress among the general population during the COVID-19 pandemic in 17 countries, around one-third of people were estimated to have elevated levels of these three psychopathological symptoms [5]. However, this meta-analysis did not analyze the reasons underlying the development of these symptoms.

In this study, it was speculated that the collective experience of overcoming the SARS outbreak in 2003 could equip Hong Kong Chinese people to develop an optimistic cognitive frame of reference towards this new pandemic (i.e., COVID-19) and that, consequently, they would be psychologically less vulnerable to its impacts. Aside from testing this hypothesis empirically, the cognitive triad model [6] was also applied to explain the mental health of Hong Kong Chinese people. Based on this theoretical model, the respondents’ self-compassion, cognitive beliefs about the valence of the pandemic, and hope were chosen as measures of their personal views about the self, the world, and the future during the onset of the pandemic in Hong Kong at the time of this study.

### 1.1. Mental Health in the COVID-19 Pandemic

An empirical research study was conducted to test the mental health states of people worldwide when facing the COVID-19 pandemic in China. The sample consisted of 4612 participants in the Philippines, Spain, Poland, Iran, the United States, Pakistan, and Vietnam. The overall mean mental health scores for all countries were as follows: depression: 1.78, anxiety: 1.90, and stress: 1.75 in a 0–3 scale per item [7]. Another study focused specifically on 678 people who were in self-quarantine in the United States, Pakistan, Canada, and the United Kingdom, in which the mean depression score was 1.37, the anxiety score was 1.20, and the stress score was 1.27 [8]. Another global study was conducted in the United States, South Korea, France, and Hong Kong, with a total of 1306 participants. The Hong Kong sample, collected from 30 March to 30 May 2020, showed that the mental health results had deteriorated more than in the other countries, with the mean score per item in depression 2.16, the anxiety score 1.35, and the stress score 2.29 [9]. This may have been because this research period captured the peak of the first wave of the pandemic in late March in Hong Kong [2]. The timing of the research period was critical in determining the participants’ psychological states because of the number of reported cases and the preventive measures enforced in different stage of pandemic.

### 1.2. Mental Health Intervention Framework—The Self, the World, and the Future

In cognition therapy, cognitive triad theory [6] highlights three root causes of mental problems: negative views about the self, about the world, and about the future. Through extracting the underlying traits and understanding negative views, intervening strategies for restoring mental health can be formulated. Beck’s main argument about depression was based on the self-view [6]. Self-view was correlated more closely with depression than either world view or future view [10]. Beck argued that negative self-schema is the core to interpreting information in a negative way, thus causing cognitive distortions [11]. However, recent empirical research on the positive cognitive triad showed that the views of both the self and the future were pivotal in predicting depression [12]. In the same study, the future view was found as the most influential in predicting life satisfaction.

This Beck cognitive triad theory was integrated into the background of positive frame of reference about having combatted SARS successfully in Hong Kong. It was anticipated that the people’s cognitive views about the self, the world, and the future would be improved in facing a similar pandemic for the general public in Hong Kong as well as the mental health.

The aim of this study was to scrutinize whether all three views could explain mental health in a new context of COVID-19, and to identify which of the three views played a more pivotal role. The study investigated the explanatory power of various cognitive triad elements to see whether the self-view was the key factor in explaining the deterioration of mental health and whether the future view was the prime factor in predicting happiness.

In this study, the general cognitive views about the self, the world, and the future were measured, respectively, by the level of self-compassion, the perception about the external environment (its positive or negative valence and degree of the impact), and the level of hope for the future.

### 1.3. Measuring Views about the Self—Self-Compassion Trait

Self-compassion is a trait that allows people to acknowledge themselves wholly and mindfully, including both their successes and their failures, with acceptance and compassion. It allows them to face themselves with understanding and kindness as they know failures are universal, and not just unique to themselves [13]. This trait allows cognitive shifting from holding onto negative events to enabling emotional regulation that protects against mental distress.

Lower perceived stress levels have been related to self-compassion [14]. They have also been related to coping methods, including positive cognitive reconstructing coping strategies and an adaptive problem-based handling method [15,16,17].

Furthermore, some researchers have found self-compassion to be a core element in mindfulness interventions that improve mental health [18,19,20]. A meta-analysis of 14 studies found a negative relationship between self-compassion and depressive, anxiety, and stress symptoms. Self-compassion is a critical, insightful factor in explaining mental health [21].

### 1.4. Measuring View of the World—Valence of COVID-19 Pandemic

Valence is the perceived positivity or negativity of an event [22]. It is also a result of the interaction between a person and an event [23]. Valence is dynamic and changeable and varies across events, individuals, and time. Kurt Lewin (1935) described positive or negative directions (valence quality) and strengths (valence quantity) of valence that depend on an individual’s needs at a particular moment. Valence or perception of the COVID-19 pandemic was related to depression in a study of mental health in Bangladesh [24].

### 1.5. Measuring View of the Future—Hope Trait

Snyder defined hope as a dispositional construct and developed the hope theory [25,26]. Hope is a belief that life goals are reachable (agency), and people have the ability and strategies (pathway) to achieve these goals. The theory evolved from cognition-based to include emotion. Hope theory states that people will perceive and ruminate information negatively and be self-critical when the hope level is low. When it is high, hope elicits positive and future orientation and accounts for a significant proportion of explanatory power in predicting optimism, psychological distress, and wellness [27]. Hope helps to reduce psychological distress, such as depressive symptoms [28,29], and to increase wellbeing [30], while self-compassion has an even higher predictive power than hope for mental health protection [31].

### 1.6. Measuring Mental Health

Symptoms of depression, anxiety, and stress were chosen as indicators of deterioration in the study participants’ psychological wellbeing. These were chosen based on an observation that they were employed as measures of mental health with community samples in a number of previous COVID-19 studies [5], and thus made cross-study comparison possible. As well as investigating people’s suffering due to the COVID-19 pandemic, this study also aimed to explore the positive side of psychological wellbeing. Happiness is a commonly employed measure of mental health in positive psychology [32], and therefore was chosen as an indicator of healthy psychological wellbeing for this study.

## 2. Aims of the Study

The study had two major goals. First, it aimed to test a hypothesis that Hong Kong people would be less vulnerable to deteriorated mental health when facing the pandemic in the early stage of COVID-19, due to a sense of optimistic frame of reference derived from the previous SARS pandemic. Specifically, it was expected that Hong Kong people would report fewer symptoms of depression, anxiety, and stress than the prevalence rates reported in published studies linked to COVID-19. Second, the study aimed to explore and compare the impact of self-compassion, valence of the COVID-19 pandemic outbreak, and hope on four selected mental health indicators (depression, anxiety, stress, and happiness). It was hypothesized that people high in self-compassion, with more positive views about the COVID-19 pandemic, and having more hope for the future would have better mental health, reporting fewer psychopathological symptoms (depression, anxiety, and stress) and feeling happier.

## 3. Method

### 3.1. Participants

Three hundred and forty five adults from Hong Kong participated. They were aged 18 or above, of Chinese ethnicity, and usually residing in Hong Kong. In Hong Kong, the ethnic composition of people living in the city is mainly Chinese (91.6%) [33], and hence this ethnic group was chosen as the focus of the study. There were 157 males and 188 females in the sample. About two-thirds of the participants were in the age groups of 36–45 years (37.4%) or 46–55 years (30.7%). Slightly more than one-tenth were in each of 26–35 years (13.3%) and 56–65 years (11.0%). Most participants had achieved a university degree or above (74%). Christianity (44.6%) was the largest religious affiliation, but almost half of the participants declared no religious affiliation (43.5%). The largest income groups were in the range of HKD 25,001–40,000 (26.7%) and HKD 70,001 or above (22.9%) per month.

### 3.2. Instruments

#### 3.2.1. Self-Compassion

Neff’s 26-item Self-Compassion Scale (SCS) was used to measure self-compassion (Neff, 2003). Three opposite matches of traits, namely, self-kindness versus self-judgment, common-humanity versus isolation, and mindfulness versus over-identification, were measured on a 5-point Likert scale. Three subscales measuring the negative end of the construct (i.e., self-judgment, isolation, and over-identification) were reverse-coded before a total SCS scale score was calculated. The scale demonstrated promising psychometric properties in Chinese populations [34,35].

#### 3.2.2. Hope

The Adult Hope Scale (AHS) (also known as the Trait Hope Scale) was used in this study [26]. This is based on Snyder’s hope theory, in which hope is defined as a positive motivation towards the future. Goal identification and achievement are two key elements central to hope. These elements are manifested as two subconstructs measured by the scale: agency (a goal-directed motivation) and pathways (a plan to reach goals). Four of the 12 items are designated to measure agency, four to measure pathways, and the remaining four items serve as distractors. Responses are made on an 8-point Likert scale. The reliability of the AHS has been reported across 17 published studies [36] as satisfactory to high (mean Cronbach’s α = 0.82).

#### 3.2.3. Valence of Event

The valence quality of COVID-19, as perceived by the participants, was measured through the question: “What effect has COVID-19 had on you?” with two response options: “good” or “bad”. The valence quantity was measured by a follow-up question: “How impactful has the recent outbreak of COVID-19 been on your life?”. A 4-point Likert scale was used, with anchors: “0 = no influence”, “1 = some influence”, “2 = moderate influence”, and “3 = great influence”. A directional “COVID-19 impact score” was computed by multiplying the coded valence quality (assign a value of +1 for “good” and −1 for “bad”) with the valence quantity score. This newly generated score ranged from −3 to +3.

#### 3.2.4. Mental Health

A 21-item short-form of the Depression, Anxiety, and Stress Scale (DASS-21) [37] was used. This was developed to measure the emotional states of non-clinical populations and to identify people who might suffer from depression, anxiety, or stress. The participants were asked to rate items describing physical and psychological states they had experienced over the preceding week, using a 4-point Likert scale (from “0 = did not apply to me” to “3 = applied to me very much or most of the time”). Three sets of 7 items were used to measure depression, anxiety, and stress scores, respectively. Severity of suffering was categorized into five levels (from normal to extremely severe) based on the summation of all 7 item scores within each of the three scales.

Aside from measuring the three aforementioned indicators of deteriorated mental health, self-descriptive happiness levels were measured by the Subjective Happiness Scale (SHS) [38] to assess the healthy side of the participants’ psychological wellbeing. The SHS is a short four-item scale; one item is reverse-coded before the computation of scale scores. In describing the development of this scale, Lyubomirsky and Lepper reported that the internal consistency reliability estimates were on the higher side (Cronbach alphas ranged from 0.79 to 0.94) [38].

All scales were translated from English into Traditional Chinese through a standard back-translation procedure [39].

### 3.3. Procedure

Before the data collection commenced, ethical approval was obtained from the human subjects ethics committee of the authors’ affiliated university (reference number B-5790-202002-14). The participants were Chinese people living in Hong Kong. The first COVID-19 case in Hong Kong was reported on 23 January 2020 and the first wave of the pandemic peaked in late March, with 1035 confirmed cases [40]. The data were collected over a period of 6 days, between 28 February 2020 and 5 March 2020, at the midpoint of the first wave of the COVID-19 outbreak in Hong Kong. In the survey period, the highly infectious and fatal nature of this new type of virus made most Hong Kong people feel anxious about personal health and safety. Face-to-face teaching in all educational institutions was suspended and social contacts were restricted. Lockdown of the city made meetings between people difficult. People were reluctant to disclose views on COVID-19 to people unknown to them, to avoid possible criticism from others. The social situation of Hong Kong society at this period of time made it infeasible to use a conventional method of collecting data through probabilistic random sampling. In quantitative research, there are multiple types of sampling methods, including random, systematic, cluster, and stratified techniques under the umbrella of probability sampling methods, and convenience, consecutive, purposive, quota, and snowball sampling techniques under the umbrella of nonprobability sampling methods. Although the probability sampling methods are more likely to generate an unbiased sample, the lack of a sampling frame to reach every Hong Kong resident made it infeasible in this study. Among various nonprobability sampling methods, techniques such as convenience sampling were not considered to be workable at the time of the pandemic outbreak in Hong Kong, as Hong Kong people were very reluctant to meet and respond to strangers at this period of time. After careful consideration of the various advantages and disadvantages of different sampling methods, it was decided that the snowball sampling technique should be adopted in this study. Major advantages of this sampling method were that (a) it would secure a fairly large sample adequate for the planned quantitative data analysis within a short period of time, and (b) it was more likely to elicit true responses from participants on sensitive issues through inherent trust of the existing network [41]. Snowball sampling has been used in a number of published studies on this topic (e.g., [42,43,44]). The questionnaire was posted to an online survey platform (Qualtrics) and was distributed through sending invitations through an instant electronic communication tool (WhatsApp) via mobile phones to recipients within the social networks of the research team. These recipients were invited to redirect the survey to their friends whenever possible to maximize the intended snowball sampling effect.

Ethical approval was obtained from the Human Subjects Ethics Sub-Committee of the author’s affiliated university. Informed consent was solicited from all respondents to ensure they had full information about the study objectives and participated on a voluntary basis. Confidentiality and anonymity were assured, and data were only collected from participants aged 18 years or above.

### 3.4. Analysis

SPSS version 25.0.0, IBM Corporation (New York, NY, USA) was used for the data analysis. Descriptive statistics were generated to examine the respondents’ mental health states and their self-views, perceptions of the impact of COVID-19, and hope for the future. Pearson correlations were calculated to examine relationships between self-compassion, valence of events, hope, and the selected mental health indicators. In addition, a regression was run to test the relative salience of self-compassion, impact of the COVID-19 pandemic, and hope as predictors of the selected mental health indicators. Type 1 error was controlled at a conventional level of α = 0.05 in all analyses.

## 4. Results

### 4.1. Descriptive Statistics

Table 1 shows the descriptive statistics for the variables used in this study (mean, SD, 95% CI of mean, skewness, and kurtosis). The means for self-compassion (M = 3.35, SD = 0.51, on a response range 1–5) and hope (M = 5.59, SD = 1.13, on a response range 1–8) were on the higher side of the response scale. For valence quality, 29% of the respondents perceived the COVID-19 pandemic as “positive”, while 71% regarded it as “negative”. For valence quantity, 69.6% rated it as having “some influence” (scored 1); and 19% as “moderate influence” (scored 2). The mean impact score was on the negative side (M =−0.68, SD = 1.15).

Table 2 shows the distribution of the three measures of deteriorated mental health, symptoms of depression, anxiety, and stress, using a categorization schedule recommended by the developer of DASS-21 [37].

From this community sample, recruited early during the outbreak of COVID-19 in Hong Kong, most reported normal levels of depression (71.3%), anxiety (67%), and stress (71%). About one-tenth reported mild levels of depression (11.6%), anxiety (8.4%), and stress (9.3%). From moderate, severe to extremely severe levels, depression, anxiety, and stress were found to be 17.1%, 24.7%, and 19.7%, respectively. Apart from referencing the five scoring levels, the mean scores for depression, anxiety, and stress were 3.35, 3.23, and 5.81, where the range of the mean scores was 0 to 20, respectively.

### 4.2. Correlations between Variables

Table 3 shows the intercorrelations between the variables chosen for this study. All mental health indicators (depression, anxiety, stress, and happiness) correlated with measures of view of the self (self-compassion), view of the world (COVID-19 impact score), and view of the future (hope) in expected directions and reached at least conventional levels of statistical significance (i.e., smaller than *p* < 0.05), Specifically, the happier respondents were those with more self-compassion, more positive views about the impact of the pandemic, or a stronger hope for the future (rs ranged from 0.20 to 0.69) and reported fewer psychopathological symptoms of depression, anxiety, and stress (rs ranged from −0.59 to −0.46). As the participants in the current sample included a large proportion of highly educated people (with bachelor degree or above) and more than half declared a religious affiliation (43.5% of participants declared no religious affiliation), these two variables were dichotomized (for educational level, participants holding a bachelor degree or above were coded as “1” and those with less education were coded as “0”; for religious affiliation, participants were coded as “1” if they had declared a religious affiliation and “0” if they had declared no religious affiliation. These two newly recoded variables did not show any significant correlation with the mental health indicators or with the three personal view variables (rs ranged from −0.13 to 0.12; *p* > 0.05).

### 4.3. Regression Analysis

To examine the relative power of self-compassion, COVID-19 impact score, and hope in predicting depression, anxiety, stress, and happiness, a series of multiple regressions was performed. In building up the regression models, self-compassion, COVID-19 impact score, and hope were entered as a set of independent variables. Taking into account the possible impacts of educational level and religious affiliation on the dependent variables, these two dichotomized variables were also entered into the regression model as control variables. In the regression model, one of the four mental health indicators (depression, anxiety, stress, and happiness) was entered as the dependent variable. Thus, four parallel models were constructed (Model 1 to Model 4). Estimates of the models were obtained by running the data on IBM SPSS version 26 using the program’s OLS procedure (Table 4).

**Figure 1:**
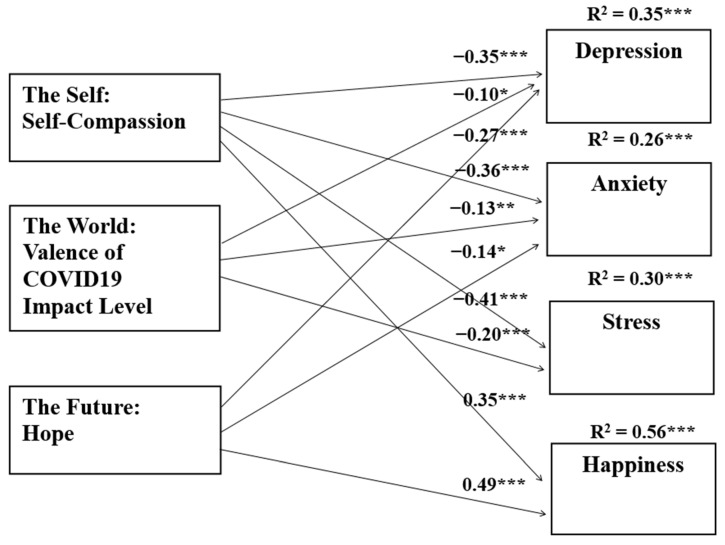
Standardized coefficients (*β*) for prediction of depression, anxiety, stress, and happiness by self-compassion, impact of COVID-19, and hope (*N* = 345). ***Note.*** * *p* < 0.05, ** *p* < 0.01, *** *p* < 0.001.

In Models 1 to 3, the three independent variables as a set did explain a substantial amount of variance in the dependent variable in the tested models (R^2^ ranged from 0.26 to 0.35). Of the three independent variables, self-compassion was the most powerful predictor in these three models (with an effect size in a weak to moderate range, β ranging from −0.35 to −0.41). In these three tested models, COVID-19 impact score and hope emerged as less salient predictors, with effect sizes in a very weak to weak range (β ranged from −0.10 to −0.27). In Model 4, hope was the most powerful predictor of happiness (β = 0.49), and self-compassion was the next most powerful (β = 0.35). Indeed, the standardized regression coefficient of happiness on hope was the largest one in the four tested models. Figure 1 summarizes the findings from these regression analyses diagrammatically.

## 5. Discussion and Conclusions

### 5.1. Mental Health of Hong Kong People and their Unique Experiences

Evidence from the surveys reported here suggests that, in the initial stage of the COVID-19 pandemic in Hong Kong, the deterioration of Hong Kong people’s mental health was not as serious as could be anticipated. Specifically, only a small portion of respondents reported moderate to extremely severe levels of depression (17.1%), anxiety (24.7%), or stress (19.7%). To better understand the mental health status of the Hong Kong people at that point in time, it is necessary to compare results of this study with those that were carried out in other populations in the same phase of the pandemic. With these criteria, a number of published studies were retrieved (e.g., [5,8,45,46,47]). Comparing with the results of a meta-analysis of 17 published studies from Asia, Europe, and North America that was conducted before May 2020 (time of early development of COVID-19 globally) and with inclusion of Depression, Anxiety, and Stress Scale (DASS) as mental health indicators, our study showed that the mental health deterioration of Hong Kong people was not as severe as for people living in these places; the meta-analysis reported an average of 33.7% heightened depressive symptoms, 31.9% heightened anxiety symptoms, and 29.6% heightened stress symptoms [5]. Findings of a cross-country empirical research conducted between late April to mid-May 2020 also showed 42%, 37%, and 25% of moderate to extremely severe levels of depression, anxiety, and stress, respectively [8].

The speculation of the common experience of SARS facilitated the early adaptation of precautionary measures, such as voluntarily mask wearing and social distancing, in responding to COVID-19 by forming a sense of control, and an initial protective psychological effect in an early pandemic stage was found and supported in a study in China [44]. The survey conducted in 190 cities in mainland China from 31 January to 2 February 2021, right after the WHO listed COVID-19 as a public health emergency, found subjects reporting 16.5% heightened depressive symptoms, 28.8% heightened anxiety symptoms, and 8.1% heightened stress symptoms [44], which shared a similar level of mental health with this study in Hong Kong.

Mental health of people changes with different phases of the pandemic. Comparing mental health with the same instrument in the same phase of pandemic development with people from different areas should be a challenging and worthwhile task. Interestingly, a cross-continental empirical study, which included Hong Kong in late March to May during the peak of first wave pandemic, revealed that the mean of the depression, anxiety, and stress score in Hong Kong was 15.1 (2.16 × 7 items in DASS Depression subscale), 9.46 (1.35 × 7 items Anxiety subscale), and 16.0 (2.29 × 7 items Stress subscale), respectively [9], which was higher than those means captured in our study before the peak of the pandemic, which were 3.35, 3.23, and 5.81, respectively. The higher depression, anxiety, and stress levels were found in Hong Kong in Dean’s study in late March 2020 [9]. This could be explained by Lam’s research when the data sampling period was capturing the peak of the first pandemic wave [2], when the reported COVID-19 cases were high and the surveillance and test, border control measures, and social distancing measures were enhanced. In addition, considering that data reported in the literature were collected at different stages of the pandemic in different locations, direct comparisons between our data and existing findings should be interpreted with caution. Consequently, support for Hypothesis 1 remains inconclusive from our data and further research is required.

### 5.2. Valence of the Outbreak of the COVID-19 Pandemic in Hong Kong

Previous research has suggested that the valence of an event can be independent of the actual nature of the event [23]. In the case of the valence quality of the COVID-19 pandemic, 29% of the respondents in this study reported its impact as “positive”. This finding can be viewed from the perspective of the valence determination principles proposed by Brendl and Higgins [23], which stated that the value attached to an event is determined mainly by whether the event can support an individual’s goal, either by itself or through its categorization. First, Hong Kong has been renowned globally for ten years for its mostly unaffordable housing prices [48], making it rather difficult to earn a living in Hong Kong. SARS in 2003 triggered a slump in the financial market and benefited property market investors dramatically, so it is logical to assume that investors and potential buyers might have viewed this COVID-19 outbreak crisis as a similar investment opportunity to purchase properties at a lower and more affordable price, which may have led to the positive valence quality.

Second, Hong Kong has the longest working hours, according to a survey comparing 17 cities worldwide [49], and this is not conducive to family life. The recent “work-from-home” measure might have been good news, enabling busy workers to have more family time.

Third, “Tao” is a traditional Chinese thought that promotes the harmonic co-existence of humans and nature as a “collective oneness”. Hence, Chinese people may view a pandemic as a messenger to awaken human beings to stop polluting our world, to restore a balance between humans and nature. From this “collective oneness” perspective, the pandemic would have a positive value in slowing down excessive and even unnecessary human activities that might destroy the balance and recovery of nature.

### 5.3. Alleviating Distresses and Promoting Happiness

A goal of this study was to test a hypothesis anchored on Beck’s cognitive triad theory [6], that negative views of oneself, the world, or the future might cause a deterioration of mental health. The context of our study was the initial stage of the outbreak of COVID-19 in Hong Kong. Specifically, self-compassion, valence towards the outbreak, and hope were chosen as measures of the respondents’ views of self, the world, and the future, respectively. As evident from the bivariate correlational analysis, all three chosen constructs showed an inverse relationship with three commonly applied indicators of psychopathological status (i.e., self-reported symptoms of depression, anxiety, and stress). Regression analyses (Model 1 to 3) further suggested that a negative view of the self (measured by a low level of self-compassion) would have more impact (as weighted by a standardized regression coefficient) on mental health deterioration than the other two views. It is also noted that hope exerted some impact on depression and the valence of the pandemic, with less influence on stress.

Our study did not focus merely on the suffering of Hong Kong people during the COVID-19 outbreak. An attempt was also made to explore how people were happier in this challenging time. Bivariate correlational analysis showed that more positive ideas about any of the three views examined in this study would promote a higher level of happiness. Multiple regression analysis indicated that hope and self-compassion exerted an influence on the promotion of happiness, with a stronger effect (as weighted by a standardized regression coefficient) from the former than from the latter variable.

### 5.4. Implications

The findings of this study offer a shred of empirical evidence to inform the formulation of effective preventive or therapeutic strategies for people who may suffer from mental distress during the global COVID-19 pandemic. In self-compassion-related education or therapy, mindfulness-based stress reduction (MBSR), mindfulness-based cognitive therapy (MBCT), and mindful self-compassion (MSC) can be some evidence-based interventions for this purpose [50,51]. Cognitive change and mindfulness elements, cognitive behavioral therapy (CBT), and mindfulness-based stress reduction (MBSR) are effective in changing the valence quality of an event from negative to positive and thus can lead to a reduction of anxiety levels [52]. Finally, future goal-oriented coaching is suitable as a hope-related intervention. The cognitive behavioral, solution-focused (CB-SF) approach was related to hope, goal-pursuing, and wellbeing [53]. This coaching intervention approach can be helpful for those seeking goal achievement and purposeful change as wellbeing enhancement [54].

Although interventions targeting changes in views of the self, the world, and the future can be valuable in alleviating negative feelings and promoting positive ones, it is imperative to set priorities given the limited resources available in Hong Kong. The findings of this study can provide some hints for the precise allocation of scarce resources. For instance, for Hong Kong people feeling unhappy and displaying some symptoms of depression, anxiety, and stress, intervention aiming at enhancing compassion towards the self should be the most cost-effective (as this variable was the most powerful predictor of depression, anxiety, and stress and the next most powerful in the prediction of happiness). However, if an ultimate goal of an intervention is to raise the happiness levels of mentally healthy people, infusing more hope would be more likely to achieve the goal (as hope was the most potent predictor of happiness). In sum, the intervention strategies should be matched to the recipients of the help.

### 5.5. Study Limitations and Future Studies

Several limitations may hinder the generalization of the findings of this study. First, due to the cross-sectional nature of the data collected in this study, the established relationship between the three views and the mental health indicators should not be considered to be causal. Second, the possibility of attaching neutral valence or giving both positive and negative valences to the COVID-19 pandemic was not tested in this study. People can experience both sides of valence quality when they have more flexibility to perceive an event as a way of coping, rather than polarizing it [55]. Third, alternative interpretations of the mental health variation in the COVID-19 pandemic observed in this sample of respondents should be considered. It should be taken into account that illness and death caused by the virus might not be the only factors involved in causing anxiety, depression, and stress, for instance, changes in lifestyle and the economic situation arising from the pandemic (e.g., lockdown and social distancing measures, high unemployment rates due to closure of enterprises). Lifestyle is measured by the number of sunlight contact hours, number of hours spent outside the living place, number of hours of exercise, and number of hours spent on entertainment, and these can be variables for future examination. As economic conditions can be measured as the amount of income change, the amount of assets, or debt change before and after the COVID-19, these can also be variables for inclusion. Furthermore, the low anxiety level reported by the participants in this study may imply that due to their customary long working hours and working under high pressure, some Hong Kong people might have welcomed the lifestyle changes brought about by the pandemic. All these speculations warrant further examination with empirical data.

As another direction for future studies, people with more diverse backgrounds should be recruited to enhance the sample representativeness and thus generalizability of the findings. People with certain sociodemographic characteristics would have different responses to the variables under consideration. For instance, highly educated people may be affected less by the pandemic (as they are less likely to lose their jobs and/or able to acquire more resources to tackle the difficulties). Likewise, people with religious beliefs are likely to have less deteriorated mental health as they can secure more spiritual and social support from their religious networks. To test the relationship between the variables more rigorously, an experimental design should be adopted to examine the causality. Last, but not least, the current practice of dichotomizing the valence of the COVID-19 pandemic was just a way to make the findings more interpretable. Further studies can explore whether more valence options should be added to better capture people’s perceptions of the crisis in reality.

## Figures and Tables

**Table 1:** Descriptive statistics of study variables (*N* = 345).

Scales/Subscales	No. of Items	α	Scale Response Range			Skew-Ness	Kurtosis
				*M (SD)*	*95% CI of Mean*		
Self-compassion	26	0.90	1–5	3.35 (0.51)	[3.30, 3.40]	−0.36	0.03
Self-kindness	5	0.75	1–5	3.41 (0.62)	[3.34, 3.48]	−0.13	−0.08
Self-judgment	5	0.73	1–5	3.29 (0.64)	[3.22, 3.36]	−0.31	−0.05
Common humanity	4	0.67	1–5	3.44 (0.66)	[3.37, 3.51]	−0.32	0.03
Isolation	4	0.79	1–5	3.36 (0.80)	[3.27, 3.45]	−0.30	−0.29
Mindfulness	4	0.73	1–5	3.59 (0.63)	[3.52, 3.66]	−0.26	0.03
Over-identification	4	0.81	1–5	3.00 (0.79)	[2.92, 3.09]	−0.38	0.26
Adult hope	8	0.93	1–8	5.59 (1.13)	[5.47, 5.71]	−0.48	0.38
Agency	4	0.88	1–8	5.45 (1.21)	[5.32, 5.58]	−0.47	0.27
Pathway	4	0.88	1–8	5.73 (1.17)	[5.61, 5.86]	−0.57	0.51
Valence quality	1	NA	−1/+1	−0.42 (0.91)	[−0.52, −0.32]	0.93	1.14
Valence quantity	1	NA	0-3	1.19 (0.61)	[1.12, 1.25]	0.79	1.42
Valence of COVID-19 Impact level ^#^	1	NA	−3–+3	−0.68 (1.15)	[−0.80, −0.56]	0.40	−0.48
DASS-21	21	0.94	0–3	12.39 (11.04)	[11.22, 13.56]	1.26	1.40
Depression	7	0.88	0–3	3.35 (4.02)	[2.93, 3.78]	1.68	2.91
Anxiety	7	0.85	0–3	3.23 (3.54)	[2.85, 3.60]	1.78	3.85
Stress	7	0.87	0–3	5.81 (4.41)	[5.34, 6.27]	0.71	0.11
Subjective happiness	4	0.83	1–7	18.70 (4.62)	[18.21, 19.19]	−0.27	−0.35

*Note*. DASS: 21-item Depression Anxiety Stress Scale. ^#^ Valence of COVID-19 impact level = valence quality (+1/−1) × valence quantity (0–3).

**Table 2:** Distribution of symptoms of depression, anxiety, and stress in the sample.

Level	Depression *N* (%)	Anxiety *N* (%)	Stress *N* (%)
0 Normal	246 (71.3%)	231 (67%)	245 (71%)
1 Mild	40 (11.6%)	29 (8.4%)	32 (9.3%)
2 Moderate	35 (10.1%)	44 (12.8%)	38 (11%)
3 Severe	12 (3.5%)	17 (4.9%)	22 (6.4%)
4 Extremely Severe	12 (3.5%)	24 (7%)	8 (2.3%)

**Table 3:** Correlations between study variables.

Variables	1	2	3	4	5	6	7
1. Self-compassion (SCS)	-	0.58 ***	0.23 ***	−0.54 ***	−0.49 ***	−0.51 ***	0.63 ***
2. Valence of COVID-19 impact level		-	0.21 ***	−0.25 ***	−0.26 ***	−0.32 ***	0.20 ***
3. Hope (AHS)			-	−0.50 ***	−0.37 ***	−0.37 ***	0.69 ***
4. Depression (DASS-D)				-	0.78 ***	0.76 ***	−0.59 ***
5. Anxiety (DASS-A)					-	0.79 ***	−0.46 ***
6. Stress (DASS-S)						-	−0.52 ***
7. Happiness (SHS)							-

*Note.* *** *p* < 0.001.

**Table 4:** Summary of regression analysis for prediction of mental health.

	Model 1			Model 2			Model 3			Model 4		
	Depression			Anxiety			Stress			Happiness		
Predictors	B	SE	β	B	SE B	β	B	SE	β	B	SE	β
Self-compassion	−0.11	0.02	−0.35 ***	−0.10	0.02	−0.36 ***	−0.14	0.02	−0.41 ***	0.12	0.02	0.35 ***
Valence of COVID-19 impact level	−0.33	0.16	−0.10 *	−0.41	0.15	−0.13 **	−0.77	0.18	−0.20 ***	0.05	0.15	0.01
Hope	−0.12	0.02	−0.27 ***	−0.05	0.02	−0.14 *	−0.05	0.03	−0.09	0.25	0.02	0.49 ***
Educational level	0.38	0.41	0.04	0.55	0.39	0.07	0.38	0.47	0.04	0.28	0.39	−0.03
Religious affiliation	−0.67	0.36	−0.08	−0.28	0.33	−0.04	−0.29	0.40	−0.03	0.48	0.34	0.05
	Model R^2^ = 0.35 ***			Model R^2^ = 0.26 ***			Model R^2^ = 0.30 ***			Model R^2^ = 0.56 ***		
	Model F (5339) = 60.09			Model F (5339) = 39.30			Model F (5339) = 48.89			Model F (5339) = 144.19		

*Note.* * *p* < 0.05, ** *p* < 0.01, *** *p* < 0.001.

## Data Availability

The data presented in this study are available on request from the corresponding author.
